# Synthesis of Fe-Co Bimetallic Supported on Cotton Residue Biochar: Catalytic Performance and Mechanistic Investigation for CO_2_ Hydrogenation

**DOI:** 10.3390/ma19194034

**Published:** 2026-09-22

**Authors:** Youzhi Yao, Tiancheng Yao, Qinghua Deng, Jiaxin Jiang, Tianyu Wang, Wenjie Wu

**Affiliations:** 1 College of Materials Engineering, Wuhu Vocational Technical University, Wuhu 241003, China; 2 College of Materials Science and Engineering, Hohai University, Changzhou 213000, China; 3 Key Laboratory of Environmental Remediation and Ecological Health, Ministry of Industry and Information Technology, School of Environmental and Biological Engineering, Nanjing University of Science and Technology, Nanjing 210094, China

**Keywords:** biochar, bimetallic, catalytic performancet, CO_2_ hydrogenation, DFT, mechanism

## Abstract

Biochar carriers were prepared from cotton residue waste, and Fe-Co bimetallic catalysts with different ratios were constructed using impregnation method. Their catalytic performance in CO_2_ hydrogenation to hydrocarbons was investigated. The structural evolution and catalytic behavior of the catalyst were systematically studied through various characterization methods such as XRD, XPS, TEM, BET, and DFT calculations. Based on the characterization of fresh and spent catalysts, the spinel phase of CoFe_2_O_4_ in the fresh catalyst is proposed to transform substantially into metallic Fe^0^ and Co^0^ upon H_2_ reduction; during subsequent CO_2_ hydrogenation, these metallic phases are proposed to partially convert to χ-(Fe_1−x_Cox)_5_C_2_ carbides, accompanied by partial graphitization of the biochar support. Because no sample was characterized after H_2_ reduction but before CO_2_ hydrogenation, this sequence is inferred. Under the same experimental conditions, Fe_15_Co_5_-BC exhibits the best CO_2_ conversion, with CH_4_ and C_2_-C_4_ alkanes as the main products, indicating that the system possesses bifunctional catalytic characteristics combining CO_2_ methanation and Fischer–Tropsch synthesis. The CO selectivity exhibited a V-shaped trend with respect to Co loading, with the lowest value achieved at the intermediate Fe/Co ratio (Fe_15_Co_5_-BC), mainly attributed to the improved overall CO_2_ conversion and the efficient conversion of carbon intermediates into hydrocarbons. DFT calculations reveal that Fe-Co bimetallic sites synergistically weaken C-O bonds and thermodynamically favor deep hydrogenation pathways. This work provides experimental and theoretical insights for the application of biochar-based bimetallic catalysts in CO_2_ hydrogenation.

## 1. Introduction

As atmospheric carbon dioxide concentrations keep climbing to unprecedented record highs, the efficient capture and utilization of CO_2_ have emerged as a core challenge to mitigate global warming and advance the circular economy. Geological CO_2_ sequestration suffers from inherent drawbacks including excessive energy consumption, leakage risks and prohibitive operational costs. In contrast, catalytic conversion that transforms CO_2_ into high-value chemicals and fuels—such as methane, methanol, dimethyl ether, formic acid and liquid hydrocarbons—boasts far broader application prospects [1]. This technical route enables long-term carbon fixation, cuts reliance on virgin fossil feedstocks, and generates economic returns to offset carbon capture expenditures. Among various CO_2_ hydrogenation pathways, methane synthesis via the Sabatier reaction (CO_2_ (g) + 4H_2_ (g) ⇋ CH_4_ (g) + 2H_2_O (g) ${\Delta G}_{298k}^{\theta }$ = −114 kJ mol^−1^ [2]) and long-chain hydrocarbon production through CO_2_ Fischer–Tropsch synthesis have attracted tremendous research attention. These processes serve as both viable carbon sequestration strategies and pivotal technologies for storing renewable energy derived from water electrolysis [3]. Such approaches deliver dual environmental benefits: they consume CO_2_, a major greenhouse gas, while storing surplus renewable power for later use [4]. Although research on CO_2_-to-hydrocarbon conversion dates back to the 1970s, its progress was long hindered by poor economic viability. In the past decade, chemical valorization of CO_2_ into high-value products has regained widespread research interest [5,6,7,8].

Catalytic hydrogenation of CO_2_ to hydrocarbons stands as a cutting-edge technology for carbon neutrality, primarily covering Power-to-Gas and Power-to-Liquid processes. These routes utilize green hydrogen and CO_2_ to produce methane-rich synthetic natural gas (SNG) or liquid hydrocarbon fuels [3]. The reaction is constrained by a trade-off between thermodynamics and kinetics. At low temperatures ranging from 200 to 350 °C, equilibrium limitations lead to low CO_2_ conversion rates. At elevated temperatures of 350–500 °C, however, side reactions proliferate, drastically lowering the selectivity toward target hydrocarbons including CH_4_ and C_2+_ species [9]. Developing promising performance catalysts represents the key to breaking this bottleneck. In conventional fixed-bed reactors, powdered catalysts suffer from poor thermal conductivity, which causes accumulated reaction heat to form localized hotspots and eventually induces sintering and deactivation of active metal sites [10,11]. While monolithic (structured) catalysts can effectively enhance heat and mass transfer within the catalyst bed [12], powdered catalysts remain widely studied thanks to their facile preparation and high operational flexibility. Biochar supports with hierarchical pore structures can not only improve the dispersion of active metal components but also alleviate localized overheating via favorable thermal conduction.

Supported metallic catalysts exhibit promising performance for CO_2_ hydrogenation within the temperature window of 200–500 °C [1]. Iron, nickel, rhodium and ruthenium are commonly adopted active metals, which are typically immobilized on supports such as metal oxides [13], zeolites [14] and commercial activated carbon [15]. Biochar, a porous carbon material fabricated via slow pyrolysis of biomass at moderate temperatures (400–500 °C), has drawn extensive research interest in catalysis due to abundant lignocellulosic waste feedstocks, relatively high carbon yields (25–45%) and outstanding sustainability [16,17]. Additionally, biochar features low production costs, abundant porous networks and rich surface oxygen-containing functional groups, rendering it an ideal standalone catalyst or catalyst support [18]. Crystalline carbon materials like carbon nanotubes (CNTs) possess readily modifiable surfaces and excellent chemical stability, and have been widely applied in catalytic research to boost metal dispersion and reactant adsorption. Nevertheless, CNTs cannot activate CO_2_ without additional promoters, and their fabrication involves high costs and unsustainable raw material supply chains. Conventional commercial activated carbon, despite its amorphous structure theoretically suited for CO_2_ hydrogenation reactions, is predominantly produced from coal, resulting in poor sustainability credentials [19]. By comparison, biochar, manufactured from agricultural and forestry residues through slow pyrolysis, acts as a more sustainable green alternative to commercial activated carbon and carbon nanotubes [20,21]. Surface functionalization of biochar—via physical/chemical activation, sulfonation, or loading of transition metals (Ni, Fe, Co, etc.)—can substantially elevate its catalytic activity [22,23]. To date, biochar has been deployed in a wide range of industrial reactions including tar reforming, biodiesel synthesis and bio-oil upgrading [24,25]. More recently, research into biochar-supported catalysts for CO_2_ hydrogenation to hydrocarbons has grown rapidly. Biochar-based catalysts are generally synthesized via impregnation with metal salt solutions, where metal ions anchor onto the support surface, followed by thermal treatment to form catalytically active metal oxides or metal carbides [26,27].

Synergistic interactions emerge between intrinsic defect sites of carbon supports and anchored metallic active phases. These interactions modulate the electronic structure of metal species, alter electron redistribution among adsorbed molecules and tune adsorption–desorption behaviors, thereby optimizing overall catalytic performance [28]. Among active metallic candidates, iron-based catalysts deliver superior activity in CO_2_ Fischer–Tropsch synthesis, while cobalt-based catalysts, well-established industrial materials, exhibit high selectivity toward long-chain hydrocarbons in traditional CO hydrogenation processes [29,30,31]. However, pure cobalt catalysts suffer from high material costs, weak CO_2_ adsorption capacity and rapid deactivation under CO_2_-rich atmospheres. Monometallic iron catalysts, by contrast, face difficulties in regulating product selectivity. Fabricating Fe-Co bimetallic catalysts has thus become a vital research strategy to combine the strong CO_2_ activation capability of iron and the superior hydrogenation selectivity of cobalt [32].

Current research on iron-based catalysts largely centers on tuning light olefin selectivity via dopants and alkali metal promoters [33,34,35]. Systematic investigations into the full product distribution (especially the proportional yields of CH_4_ and C_2+_ hydrocarbons) over biochar-supported Fe-Co bimetallic catalysts during CO_2_ hydrogenation remain scarce. Existing research has confirmed that cobalt doping can enhance the adsorption capacity of carbon dioxide and promote the formation of active iron carbide phases, which can facilitate carbon–carbon coupling reactions on the carbide surface [36]. In this work, we fabricated Fe-Co bimetallic catalysts supported on cotton residue-derived biochar and explored their catalytic performance for CO_2_ hydrogenation to hydrocarbons, with particular focus on how cobalt loading modulates product distribution. Composite catalysts were prepared by impregnating cotton residue biochar with Fe(NO_3_)_3_·9H_2_O and Co(NO_3_)_2_·6H_2_O at varying mass ratios, followed by reduction under a hydrogen atmosphere. Among the three catalyst formulations tested at 320 °C, Fe_15_Co_5_-BC achieved the highest CO_2_ conversion and methane selectivity, alongside substantial co-production of C_2_-C_4_ hydrocarbons. This study validates that cotton residue can serve as an eco-friendly, low-cost support for Fe-Co bimetallic catalysts applied in thermocatalytic CO_2_ hydrogenation. Furthermore, density functional theory (DFT) calculations were adopted to resolve the full reaction pathway at the atomic scale, elucidating the structure–activity relationships between key reaction intermediates and active sites. These findings provide theoretical guidance for the rational design of high-efficiency CO_2_ hydrogenation catalysts.

## 2. Experimental Section

### 2.1. Preparation of Catalytic Materials

The Fe(NO_3_)_3_·9H_2_O and Co(NO_3_)_2_·6H_2_O used in this study were purchased from Sinopharm Chemical Reagent Co., Ltd. (Shanghai, China) and were of analytical grade. The deionized water had a resistivity of 18 MΩ·cm.

The cotton residue was crushed with a grinder (Test FW80, Shanghai Jinpeng Analytical Instrument Co., Ltd., Shanghai, China) for 5 min and sieved to <0.5 mm (35 mesh). It was then washed three times with deionized water and dried at 105 °C before pyrolysis. Subsequently, 5.0272 g of the dried material was weighed, placed into a crucible, compacted, covered, and transferred to a muffle furnace. Calcination proceeded from an initial temperature of 30 °C at a heating rate of 5 °C/min for 120 min, reaching a holding temperature of 600 °C maintained for 60 min. This process yielded 2.1415 g of biochar, corresponding to a yield of approximately 42.5%, which is comparable to or higher than the yields reported for some other feedstocks under similar pyrolysis conditions [37].

Active Fe-Co species with Fe/Co molar ratios of 15:1, 15:5 and 15:9 were loaded onto biochar via the wet impregnation method, with the biochar amount kept constant across all preparations. The detailed synthetic procedures are presented below: Appropriate amounts of Fe(NO_3_)_3_·9H_2_O and Co(NO_3_)_2_·6H_2_O were weighed based on the preset Fe/Co molar ratios of 15:1, 15:5 and 15:9, then transferred into a small beaker. After adding 10 mL of deionized water, the mixture was ultrasonicated until the metal salts dissolved completely. A measured portion of biochar was placed in a larger beaker. The prepared metal salt solution was added dropwise to the biochar under constant stirring with a glass rod, while supplementary deionized water was introduced simultaneously until the biochar reached its saturated water absorption limit. The slurry aged at ambient temperature for 60 min to ensure uniform impregnation. After impregnation, the mixture was oven-dried at 101 °C for 120 min to remove residual water. The dried solid was transferred to a covered crucible and subjected to calcination in a muffle furnace. The temperature schedule was configured as follows: starting from an initial temperature of 30 °C, the furnace was ramped up at a rate of 5 °C/min for 100 min until reaching the target calcination temperature of 550 °C, which was maintained for a 120 min isothermal hold. The as-obtained calcined sample displayed a pale reddish-brown surface.

For control experiments, we prepared monometallic Fe-only (Fe_15_-BC) and Co-only (Co_15_-BC) catalysts using the same method. We also prepared a physical mixture of Fe_15_-BC and Co_15_-BC (Fe:Co molar ratio = 1:1) by gently grinding the two monometallic catalysts together in an agate mortar for 30 min. The bare biochar carrier served as a blank control, without any metal loading.

The actual metal loading of the prepared catalysts (including single-metal control samples) was determined using inductively coupled plasma optical emission spectrometry (ICP-OES, PerkinElmer Optima 8300, PerkinElmer, Inc., Wellesley, MA, USA). Prior to analysis, each catalyst sample (approximately 0.05 g) was digested in a mixture of concentrated nitric acid and hydrochloric acid (volume ratio 3:1) at 180 °C for 30 min using a microwave digestion system (CEM Mars 6, CEM Corporation, Matthews, NC, USA). Subsequently, the digested solution was diluted to 50 mL with deionized water and measured. The ICP-OES results are summarized in Table S1. The total metal loading varies with the Fe/Co ratio, increasing from 15.2 wt% to 19.1 wt% as Co incorporation rises, while the Fe loading correspondingly decreases.

### 2.2. Catalytic Activity Testing

CO_2_ hydrogenation was conducted in a stainless-steel tubular fixed-bed reactor with an inner diameter of 6.0 mm (as illustrated in Figure 1). Each test used 0.2500 g of catalyst (after calcination, before reduction) for catalytic performance evaluation. Prior to the reaction, the catalyst was reduced in a pure hydrogen flow of 40 sccm at 400 °C for 240 min. After reduction, we cooled the reactor to 320 °C and introduced a mixed reaction gas of CO_2_/H_2_/Ar (19.3/77.2/3.5 vol%), gradually increasing the system pressure to 2.0 MPa.

The effluent gas from the fixed-bed reactor was analyzed using an online gas chromatograph (GC-2014, Shimadzu, Kyoto, Japan) equipped with both a thermal conductivity detector (TCD) and a flame ionization detector (FID). The TCD carrier gas was He, and Ar in the feed (3.5 vol%) was used as an internal standard to correct for total flow changes. CO and CO_2_ were separated using a Porapak Q column (Waters Corporation, Milford, MA, USA) (3 m × 3 mm) and quantified by TCD with He as the carrier gas. Hydrocarbon products were separated using an HP-PLOT Q capillary column (Agilent Technologies, Santa Clara, CA, USA) (30 m × 0.32 mm × 20 μm) and quantified by FID. The C_5+_ fraction was collected in a cold trap (0 °C) and analyzed offline by gas chromatography-mass spectrometry (GC-MS, Agilent 7890B-5977B, Agilent, Santa Clara, CA, USA). Five-point external calibration was performed using certified standard gas mixtures containing CO_2_, CO, CH_4_, C_2_H_4_, C_2_H_6_, C_3_H_6_, C_3_H_8_, C_4_H_8_, and C_4_H_10_. The calibration ranges were 0.1–30 vol% for CO_2_ and CH_4_, 0.05–10 vol% for CO, and 0.01–5 vol% for C_2_-C_4_ species. Ar (3.5 vol%) was used as an internal standard. Sampling frequency was every 30 min during the 240 min reaction.

The CO_2_ conversion, CO selectivity, total hydrocarbon selectivity, and individual hydrocarbon selectivities were calculated using Equations (1)–(4), with Ar as the internal standard [38,39]:

(1) $$X_{CO_{2}}=\frac{{\left(C_{CO_{2}}/C_{Ar}\right)}_{in}-{\left(C_{CO_{2}}/C_{Ar}\right)}_{out}}{{\left(C_{CO_{2}}/C_{Ar}\right)}_{in}}\times 100\%$$

(2) $$S_{CO}=\frac{{\left(C_{CO}/C_{Ar}\right)}_{out}}{{\left(C_{CO_{2}}/C_{Ar}\right)}_{in}-{\left(C_{CO_{2}}/C_{Ar}\right)}_{out}}\times 100\%$$

(3) $$S_{TotalHC}=100\%-S_{CO}$$

(4) $$S_{HC,i}^{\left(HC\text{-}normalized\right)}=\frac{n_{i}{\left(C_{HC,i}/C_{Ar}\right)}_{out}}{\sum_{j}n_{j}{\left(C_{HC,j}/C_{Ar}\right)}_{out}}\times 100\%$$

where $X_{CO_{2}}$ is the CO_2_ conversion, $S_{CO}$ is the CO selectivity, $S_{TotalHC}$ is the total hydrocarbon selectivity, $S_{HC,i}^{\left(HC\text{-}normalized\right)}$ represents the percentage of individual hydrocarbons within the total hydrocarbon fraction, $C_{CO_{2}}$, $C_{CO}$, $C_{HC,i}$, and $C_{Ar}$ are the measured concentrations (vol%) of the corresponding species, $n_{i}$ is the carbon number of hydrocarbon i, and subscripts “in” and “out” denote the reactor inlet and outlet, respectively.

Hydrocarbon yield was calculated as [38,39]:

(5) $$Y_{HC}=X_{CO_{2}}\times S_{TotalHC}/100$$

Carbon balance was calculated as [38,39]:

(6) $$Carbon\;balance=\frac{{\left(C_{CO}/C_{Ar}\right)}_{out}+\sum_{i}n_{i}{\left(C_{HC,i}/C_{Ar}\right)}_{out}}{{\left(C_{CO_{2}}/C_{Ar}\right)}_{in}-{\left(C_{CO_{2}}/C_{Ar}\right)}_{out}}\times 100\%$$

The total hydrocarbon selectivity was calculated as 100%-CO selectivity because no oxygenates (e.g., methanol, ethanol, aldehydes) were detected by GC-MS, and the carbon balance was 97.6–98.6% for all runs. Carbon deposition was not directly quantified and is acknowledged as a limitation.

### 2.3. Characterization

The crystal structure was analyzed by X-ray diffraction (XRD) on a Bruker D8 ADVANCE diffractometer with Cu-Kα radiation (λ = 0.15406 nm) over a 2θ range of 10–80° at a scanning rate of 0.05°/s. The functional groups of the catalysts were characterized using a Thermo Nicolet iS5 Fourier transform infrared (FTIR) spectrometer (Thermo Fisher Scientific, Waltham, MA, USA). The chemical states of surface elements were analyzed by X-ray photoelectron spectroscopy (XPS) on a Thermo Scientific Escalab 250xi spectrometer (Thermo Fisher Scientific, Waltham, MA, USA). All spectra were fitted using Avantage 6.9.0 software, and the binding energies were calibrated by referencing the C 1s peak of adventitious carbon at 284.8 eV. The morphology, particle size distribution, and lattice fringes of the catalysts were observed using an FEI Talos F200X transmission electron microscope (TEM) (FEI, Lausanne, Switzerland), and the crystal structure was analyzed in conjunction with selected area electron diffraction (SAED). The specific surface area and pore structure were measured using an ASAP 2460 physisorption analyzer (Micromeritics Instrument Corporation, Norcross, GA, USA). Prior to analysis, the samples were degassed under vacuum at 200 °C for 6 h, followed by N_2_ adsorption–desorption measurements at −196 °C. The specific surface area was calculated using the Brunauer–Emmett–Teller (BET) equation, while the pore size distribution and pore volume were determined by the Barrett–Joyner–Halenda (BJH) method.

The detailed structural and spectroscopic characterizations (XRD, XPS, TEM, HRTEM, SAED, and FTIR) were focused on the three bimetallic catalysts, while the bare biochar support, monometallic controls, and physical mixture were primarily evaluated for catalytic performance and textural properties.

## 3. Results and Discussion

### 3.1. Phase Analysis of Fresh-Catalysts

XRD characterization was performed on the three as-prepared fresh catalysts to identify their structural features (Figure 2a). Fe_15_Co_1_-BC shows diffraction signals at 24.1°, 33.1°, 35.6°, 40.8°, 49.5°, 54.1°, 57.5°, 62.4°, and 64.0°. Given the low cobalt loading, these peaks match the standard α-Fe_2_O_3_ pattern (PDF#33-0664), corresponding to the (012), (104), (110), (113), (024), (116), (122), (214), and (300) lattice planes. The XRD profiles change systematically upon cobalt incorporation. As the Co proportion increases from 1 to 9, the characteristic α-Fe_2_O_3_ diffraction peaks gradually diminish and eventually vanish, while peaks assigned to the CoFe_2_O_4_ spinel phase emerge and intensify progressively. The major diffraction peaks of Fe_15_Co_9_-BC located at 30.1°, 35.5°, 43.4°, 57.2° and 62.7° were consistent with the standard CoFe_2_O_4_ reference (PDF#03-0864), indexed to the (220), (311), (400), (511) and (440) planes, consistent with the cubic spinel structure being the dominant crystalline phase of the catalyst. With increasing Co content, the XRD peaks become slightly broader, which may reflect increased lattice strain and compositional heterogeneity associated with Co incorporation into the Fe oxide lattice. The transformation from α-Fe_2_O_3_ to CoFe_2_O_4_ is associated with progressive incorporation of Co into the Fe oxide lattice. As indicated by XPS below, Co exists as a mixture of Co^2+^ and Co^3+^. The formation of CoFe_2_O_4_ likely involves Co^2+^ occupying octahedral sites, but the exact mechanism is complex and cannot be resolved solely from the present data. Further investigation is required to clarify the role of Co^2+^/Co^3+^ in the spinel formation. This observation aligns with the phase evolution behavior reported for Fe-Co oxide systems [32,40].

To probe the bonding environments suggested by the XRD phase evolution, FTIR spectra were collected. FTIR spectra of all samples were collected via the ATR mode. Prior to measurement, each sample was vacuum-dried at 100 °C for 30 min, and all tests were carried out under continuous nitrogen purge to eliminate spectral interference from adsorbed water on hydroxyl and carbonyl bands; the resultant spectra are displayed in Figure 2b. All fresh catalysts exhibit a broad, intense absorption band centered at 3420 cm^−1^, which we assign to the stretching vibration of abundant surface hydroxyl groups (-OH) on the biochar. The band near 2930 cm^−1^ primarily stemmed from asymmetric stretching vibrations of aliphatic CH_2_ and CH_3_ moieties. The distinct peak at 1590 cm^−1^ arose from C=O carbonyl stretching, with minor contributions from conjugated C=C double bonds [41]. The absorption signal at 1230 cm^−1^ corresponded to the stretching vibration of C-OH groups [42]. Furthermore, a characteristic band originating from metal–oxygen (M-O) bond vibrations was detected at approximately 580 cm^−1^ [43]. As Co loading increases, the M-O band shifts progressively from 553 cm^−1^ (Fe_15_Co_1_-BC) to 562 cm^−1^ (Fe_15_Co_5_-BC) and 578 cm^−1^ (Fe_15_Co_9_-BC), corresponding to a total shift of 25 cm^−1^ toward higher wavenumbers. This trend is consistent with altered Fe-O-Co bonding environments and cross-validates the phase evolution deduced from XRD, although FTIR alone does not provide conclusive evidence for Fe-O-Co bond formation.

To further resolve the surface chemical states and oxidation states of Fe and Co, XPS measurements were performed. X-ray photoelectron spectroscopy (XPS) measurements were conducted to unravel the evolution of active sites over the catalysts during CO_2_ hydrogenation. The XPS tests were performed using an Al-Kα X-ray source with a pass energy of 20 eV and constant step-size scanning, to analyze the surface chemical states of fresh catalysts [44].

The C 1s spectrum (Figure 3a) displayed three characteristic peaks at 284.8 eV, 286.3 eV and 288.5 eV, which correspond to graphitic C-C bonds, C-O bonds and O-C=O groups, respectively. This confirms the abundant oxygen-containing functional groups distributed on the biochar support surface. In the O 1s spectrum (Figure 3b), the broad signal ranging from 531.5 eV to 532.0 eV originates from metal–oxygen–carbon (M-O-C) linkages. This demonstrates that oxygenated functional groups act as anchoring sites to coordinate with Fe and Co species, and such metal-support interactions effectively stabilize highly dispersed metallic nanoparticles.

For the Fe 2p spectrum (Figure 3c), the dominant Fe 2p_3/2_ peak centered at 711.5 eV, accompanied by distinct satellite peaks at 719 eV and 733 eV, represents the fingerprint signature of Fe^3+^ cations [45,46]. This reveals that iron predominantly exists as Fe_2_O_3_, while partial Fe^3+^ ions are incorporated into mixed-valence spinel lattices. The Co 2p spectrum (Figure 3d) shows characteristic peaks at 780.2 eV (Co 2p_3/2_) and 795.5 eV (Co 2p_1/2_). Combined with its satellite peak features, this verifies that cobalt species coexist in divalent and trivalent oxidation states (Co^2+^/Co^3+^) [47].

Collectively, these XPS results demonstrate that Fe and Co species are highly dispersed on the biochar surface in oxidized forms within fresh catalysts. These oxidized precursors provide a favorable structural foundation for the in situ generation of metallic active sites (Fe^0^/Co^0^) and metal carbides during the subsequent H_2_ reduction treatment.

To visualize the morphological consequences of the chemical states revealed by XPS, TEM and HRTEM were employed. The microstructural features of fresh catalysts were further characterized by transmission electron microscopy (TEM), high-resolution transmission electron microscopy (HRTEM) and selected-area electron diffraction (SAED), with the results presented in Figure 4. Nanoparticles of all three catalysts exhibited irregular spherical morphologies (Figure 4a,d,g). Their statistically averaged particle sizes were measured as 16.1 ± 2.5 nm, 18.7 ± 2.7 nm and 28.1 ± 4.2 nm with increasing Co loading, which indicates that the introduction of Co exerts a remarkable effect on catalyst particle size. It should be noted that the total metal loading also increases with Co incorporation (from 15.2 to 19.1 wt%), which may contribute to the observed increase in particle size. HRTEM images (Figure 4b,e,h) revealed characteristic lattice fringes of 0.251 nm and 0.162 nm across all samples, which are assigned to the (311) and (511) crystal planes of CoFe_2_O_4_, respectively. Distinct diffraction rings corresponding to the (311) plane of CoFe_2_O_4_ could be clearly identified in the SAED patterns (Figure 4c,f,i). As the Co proportion increased, inner diffraction rings belonging to the (220) plane emerged inside the (311) ring, verifying the polycrystalline nature of all three samples [48]. These observations are consistent with the XRD findings. For Fe_15_Co_1_-BC, α-Fe_2_O_3_ remains the dominant crystalline phase, and CoFe_2_O_4_ becomes dominant only at higher Co loadings (Fe_15_Co_5_-BC and Fe_15_Co_9_-BC). The HRTEM and SAED results therefore support the progressive α-Fe_2_O_3_ → CoFe_2_O_4_ transformation rather than indicating that CoFe_2_O_4_ is the sole dominant phase in all fresh catalysts. Such a structure lays a solid structural basis for the evolution of catalytically active species during the subsequent hydrogen reduction process.

### 3.2. Catalytic Reactivity of the Catalysts

After reduction in pure hydrogen, we collected catalytic data over 240 min at 320 °C, 2 MPa, a gas hourly space velocity (GHSV) of 6000 mL·g^−1^·h^−1^, and an H_2_/CO_2_ molar ratio of 4:1. The CO_2_ conversion, CO selectivity, total hydrocarbon selectivity, and individual hydrocarbon selectivities were calculated using Equations (1)–(4), with Ar in the feed (3.5 vol%) as an internal standard to correct for total flow changes. Carbon balance and hydrocarbon yield were calculated using Equations (5) and (6), respectively, and all runs gave carbon balances of 97.6–98.6%. Their CO_2_ hydrogenation catalytic performance was evaluated. For comparison, iron-only (Fe_15_-BC), cobalt-only (Co_15_-BC), a physical mixture of Fe_15_-BC and Co_15_-BC (molar ratio of 1:1), and a bare biochar carrier were also evaluated under the same conditions; the conversion rate of CO_2_ on the bare carrier was negligible (<1%), and thus it was not included in the main comparison. Detailed catalytic data are summarized in Table 1.

As the Fe/Co molar ratio decreases (i.e., with increasing Co incorporation), the CO_2_ conversion rises from 37.8% to 49.9%, then declines slightly to 44.7%. The CO selectivity exhibits a V-shaped trend: 27.2% (Fe_15_Co_1_-BC) → 19.6% (Fe_15_Co_5_-BC) → 28.8% (Fe_15_Co_9_-BC). Correspondingly, the total hydrocarbon selectivity follows the inverse trend: 72.8% → 80.4% → 71.2%, calculated as 100%-CO selectivity because no oxygenates were detected by GC-MS and the carbon balance was 97.6–98.6%. (see Table 1 and Supplementary Table S1).

Within the hydrocarbon fraction (normalized to 100%), CH_4_ selectivity rises from 38.5% to a maximum of 56.2%, then drops to 46.7%, whereas C_2_-C_4_ alkane selectivity remains roughly constant at approximately 40%. As Co loading increases, the selectivities of C_2_-C_4_ alkenes, C_5+_ alkenes, and C_5+_ alkanes all decrease, mirroring the CH_4_ trend.

The control experiments further support the Fe-Co synergy, although the Fe/Co ratio, total metal loading, and particle size vary simultaneously and cannot be fully decoupled. The CO_2_ conversion rate of Fe_15_-BC was 28.3%, the CO selectivity was 44.5%, and the CH_4_ selectivity was 18.7%. For Co_15_-BC, the conversion rate was 32.1%, the CO selectivity was 41.2%, and the CH_4_ selectivity was 22.3%. The physical mixture of Fe_15_-BC and Co_15_-BC exhibited intermediate performance (with a conversion rate of 35.6%, a CO selectivity of 38.7%, and a CH_4_ selectivity of 25.1%). The physical mixture has an overall Fe/Co molar ratio of 1:1, which differs from the 3:1 ratio of Fe_15_Co_5_-BC, which serves only to test whether simple mixing produces synergy. The bimetallic Fe_15_Co_5_-BC catalyst substantially outperforms all controls.

To assess short-term steady operation, time-on-stream (TOS) data for Fe_15_Co_5_-BC were collected over the 240 min reaction period (Supplementary Table S2). Both CO_2_ conversion and CH_4_ selectivity reached steady state within 120 min and remained stable throughout the remaining 120 min, with average values of 49.9 ± 0.5% and 56.2 ± 0.6%, respectively, during the 120–240 min steady-state window. The reported errors represent the standard deviation of steady-state time points (120–240 min) from a single run; independent replicate experiments were not performed. This stabilization time is consistent with literature reports. Bukur et al. showed that H_2_-reduced precipitated iron Fischer–Tropsch catalysts reach steady-state activity within 2–4 h [38]. Yuan et al. reported that Fe-Co bimetallic CO_2_ hydrogenation catalysts typically exhibit an induction period of approximately 3 h before reaching steady state at about 4 h on stream [39]. These results confirm that the catalytic data reported in Table 1 reflect steady-state performance, within the 240 min test, rather than transient behavior. However, the 240 min test demonstrates short-term steady operation only and should not be presented as evidence of long-term catalyst stability. Long-term stability testing (>500 h) under industrial conditions was not performed in this work and remains an important direction for future investigation.

The decrease in CO selectivity at moderate Co loading likely arises primarily from the increased overall CO_2_ conversion and the more efficient conversion of carbon intermediates to hydrocarbons, rather than from direct inhibition of the reverse water–gas shift (RWGS) reaction. Co incorporation also appears to accelerate CO_2_ activation and the subsequent hydrogenation of reaction intermediates. The synergistic effect between Fe and Co regulates the product distribution, promoting the preferential conversion of carbon species to CH_4_ and short-chain C_2_-C_4_ alkanes.

As Co loading increases, the total metal loading also increases (from 15.2 wt% to 19.1 wt%), while the Fe loading decreases. The average particle size also increases from 16.1 to 28.1 nm. The observed performance differences thus reflect the combined effects of Co incorporation, varying total metal loading, changing Fe/Co ratio, and changing particle size. A simple metal-normalized conversion (CO_2_ conversion divided by total metal loading) gives 2.49, 2.92, and 2.34% per wt% metal for Fe_15_Co_1_-BC, Fe_15_Co_5_-BC, and Fe_15_Co_9_-BC, respectively, supporting the optimal intermediate composition. However, this normalization does not account for metal dispersion or surface exposure, and turnover frequencies were not determined. Control experiments support that the bimetallic composition is essential for enhanced performance, though the precise contribution of each factor requires further systematic investigation. The mechanistic interpretation in this work focuses on the electronic synergy between Fe and Co at bimetallic sites, as suggested by DFT calculations, rather than attributing all performance differences solely to Co incorporation. Among all tested Fe/Co ratios, Fe_15_Co_5_-BC gives the highest CH_4_ selectivity within the hydrocarbon fraction, at 56.2%. C_2_-C_4_ light alkanes nevertheless account for nearly 40% of the hydrocarbon products, suggesting that this catalyst system exhibits bifunctional activity combining CO_2_ methanation with Fischer–Tropsch synthesis, rather than behaving as a purely selective methanation catalyst.

### 3.3. Phase Analysis of the Spent-Catalysts

The structural evolution of the spent catalysts was investigated by combining XRD, XPS, HRTEM/SAED, Raman, and FTIR, with each technique providing complementary information on the phase transformation and surface changes. Because no sample was characterized after H_2_ reduction but before CO_2_ hydrogenation, the assignment of structural changes to the reduction stage versus the reaction stage is inferred rather than directly observed. The spent catalyst data therefore represent the cumulative result of both stages.

Figure 5a shows the XRD patterns of spent catalysts. The characteristic reflections of the CoFe_2_O_4_ spinel phase are largely absent after reaction, indicating that the spinel precursor is substantially reduced and transformed into new phases. A new diffraction signal emerges at 43.8° (2θ), which coincides with the principal peak of Fe_5_C_2_ (PDF#51-0997). As Co loading increases, the 43.8° diffraction peak shifts slightly toward higher 2θ angles, consistent with partial substitution of Fe sites in the Fe_5_C_2_ lattice by Co atoms to form a χ-(Fe_1−x_Co_x_)_5_C_2_ solid solution [49,50,51]. The peak broadening observed in the fresh catalysts becomes less pronounced after reaction, likely because the transformation to metallic and carbide phases relieves part of the lattice strain.

XPS was used to track the chemical-state changes associated with this phase transformation. In XPS (Figure 6c), a feature at approximately 707.5 eV is consistent with iron carbides [52]; however, Fe 2p spectra are complicated by multiplet splitting, satellite features, and overlapping contributions from Fe^0^, Fe^2+^, Fe^3+^, and carbide species [45,46,47]. In the Fe 2p spectra, a distinct new peak at 706.8 eV corresponds to metallic iron (Fe^0^), while the shoulder at approximately 707.5 eV corresponds to the iron carbide species. The characteristic Fe^3+^/Fe^2+^ oxide peaks weaken substantially but do not disappear entirely, implying that some iron species may be encapsulated by the carbon support or form strong metal-support interactions that resist complete reduction. In the Co 2p spectra, a new peak at 778.1 eV is characteristic of metallic cobalt (Co^0^) [47], whereas partial Co^2+^ signals remain detectable. Previous work has demonstrated a synergistic interplay between Co^0^ and Co^2+^: Co^0^ sites dominate H_2_ activation and dissociation, while adjacent Co^2+^ sites accelerate CO_2_ adsorption and activation [53].

HRTEM and SAED provided direct structural evidence for the coexistence of metallic and carbide phases. HRTEM imaging reveals lattice fringes at approximately 0.205 nm, which we index to the (510) plane of χ-Fe_5_C_2_ [50] (Figure 7c,f,i). SAED patterns also show diffraction rings corresponding to this phase (Figure 7b,e,h). Lattice fringes with an interplanar spacing of 0.203 nm match the (110) plane of metallic Fe^0^ (PDF#87-0721), suggesting that some iron species retain the zero-valent state after reaction. Additionally, lattice fringes of 0.350 nm assigned to the (002) plane of graphite microcrystals are observed for Fe_15_Co_5_-BC, suggesting partial graphitization of the biochar support driven by thermal effects and catalytic graphitization mediated by metallic nanoparticles [54,55,56,57].

Raman spectroscopy further supports the graphitization of the biochar support. Taking Fe_15_Co_5_-BC as a typical example, its Raman spectrum is shown in Supplementary Figure S1. After peak fitting, the I_D_/I_G_ area ratio decreases from 2.14 (fresh) to 1.98 (spent), and the I_D_/I_G_ height ratio decreases from 0.88 to 0.70, consistent with the HRTEM observation of 0.350 nm graphite fringes and with the attenuation of C-O and OC=O signals in XPS. Together, these results indicate partial graphitization of the biochar support during reaction. Collectively, these complementary techniques provide strong evidence for the formation of a Co-substituted χ-carbide phase. Definitive confirmation would ideally require additional techniques such as Mössbauer spectroscopy or synchrotron XRD. Nevertheless, the present multi-technique evidence strongly supports this assignment. All three spent catalysts thus undergo substantial crystal structural transformation during reaction, with the spinel precursor largely reduced and converted to metallic and carbide phases.

FTIR was used to examine the changes in surface functional groups and metal–oxygen bonds. Compared with the fresh catalysts, the spent samples show little change in major FTIR absorption band positions, yet their peak intensities vary noticeably (Figure 5b). This indicates that the types of surface functional groups on the biochar support remain largely preserved during catalysis, though their relative abundances change. The absorption intensities of the -OH (3420 cm^−1^), C=O (1590 cm^−1^), and C-OH (1230 cm^−1^) bands decline moderately, suggesting that oxygen-containing functional groups are gradually consumed or transformed during reduction and reaction. The originally sharp metal–oxygen (M-O) vibrational band at 580 cm^−1^ attenuates and broadens considerably, consistent with the reduction in iron and cobalt oxides. This inference is consistent with the XRD and XPS results.

Definitive confirmation would ideally require additional techniques such as Mössbauer spectroscopy or synchrotron XRD. Nevertheless, the present multi-technique evidence strongly supports this assignment. All three spent catalysts thus undergo substantial crystal structural transformation during reaction, with the spinel precursor largely reduced and converted to metallic and carbide phases. No sample was characterized after H_2_ reduction but before CO_2_ hydrogenation. Therefore, the exact extent to which reduction and carburization occur during the H_2_ pretreatment versus during the CO_2_ hydrogenation reaction cannot be fully deconvoluted from the present data. The proposed sequence—spinel → metallic Fe^0^/Co^0^ → χ-carbide—is consistent with the known reducibility of CoFe_2_O_4_ and with literature reports on similar Fe-Co systems, but it should be regarded as an inferred pathway rather than a directly observed one.

The XPS spectra of the spent catalysts provide further insight into the surface chemical states. In the C 1s spectra (Figure 6a), the signal intensities of oxygen-containing functional groups (C-O and O-C=O) attenuate markedly, indicating partial removal or reduction in these groups on the biochar surface under reaction conditions. This observation is consistent with the partial graphitization indicated by the decreased I_D_/I_G_ ratio in Raman spectroscopy and the 0.350 nm graphite fringes observed by HRTEM. The O 1s spectra (Figure 6b) show a drastic decline in the signal assigned to metal–oxygen bonds (530.0 eV), further consistent with the reduction in metal oxides and with the disappearance of the CoFe_2_O_4_ spinel reflections in the XRD patterns of the spent catalysts.

In the Fe 2p spectra (Figure 6c), a distinct new peak at 706.8 eV corresponds to metallic iron (Fe^0^), while the shoulder at approximately 707.5 eV is consistent with the iron carbide species discussed above [52]. The characteristic Fe^3+^/F^2+^ oxide peaks weaken substantially but do not disappear entirely, implying that some iron species may be encapsulated by the carbon support or form strong metal-support interactions that resist complete reduction. This residual oxidized iron is consistent with the incomplete transformation of the spinel precursor inferred from the combined XRD, XPS, and HRTEM results. In the Co 2p spectra (Figure 6d), a new peak at 778.1 eV is characteristic of metallic cobalt (Co^0^) [47], whereas partial Co^2+^ signals remain detectable. Previous work has proposed a synergistic interplay between Co^0^ and Co^2+^, in which Co^0^ sites dominate H_2_ activation and dissociation while adjacent Co^2+^ sites accelerate CO_2_ adsorption and activation [53]. Although this interpretation is supported by the present Co 2p data, further dedicated experiments would be required to establish the precise role of each cobalt species under reaction conditions.

To visualize the morphological and structural consequences of the chemical-state changes described above, TEM, HRTEM, and SAED were performed. TEM images (Figure 7a,d,g) reveal that the interfacial boundary between metallic nanoparticles and the biochar support becomes significantly blurred compared with fresh catalysts, which is likely due to lattice distortion from redox cycling and strong metal-support interaction between the bimetallic nanoparticles and the carbon matrix, as suggested by the XPS and XRD results. Some Fe-Co components may also infiltrate the biochar pore channels during catalysis, although this is inferred rather than directly observed.

HRTEM images (Figure 7c,f,i) further indicate the coexistence of multiple crystalline phases in all three spent catalysts. Lattice fringes with an interplanar spacing of 0.203 nm match the (110) plane of metallic Fe^0^ (PDF#87-0721), suggesting that some iron species retain the zero-valent state after reaction, consistent with the Fe 2p XPS results. Consistent with the carbide phase identified above, lattice fringes at approximately 0.205 nm are assigned to the (510) plane of χ-(Fe_1−x_Co_x_)_5_C_2_ [50]. Additionally, lattice fringes of 0.350 nm assigned to the (002) plane of graphite microcrystals are observed for Fe_15_Co_5_-BC. This graphitization may facilitate electron transfer between metallic active sites and the carbon support. The SAED patterns (Figure 7b,e,h) also show diffraction rings consistent with the carbide phase, providing complementary structural evidence. Because no sample was characterized after H_2_ reduction but before CO_2_ hydrogenation, the assignment of these phases to the reduction versus reaction stages remains inferred. The potential impact of particle sintering and continuous structural evolution on catalyst lifetime warrants systematic investigation in future work.

We also characterized the pore structures by N_2_ adsorption–desorption isotherms (Figure 8). All samples exhibit Type IV isotherms with H4 hysteresis loops, indicating the coexistence of microporous and mesoporous structures [58]. The rapid N_2_ uptake at low relative pressure (P/P_0_ < 0.02) indicates abundant micropores, while the H4 hysteresis loop at higher P/P_0_ suggests slit-shaped mesopores [59]. The H4-type hysteresis loops observed here are characteristic of micro-mesoporous carbon materials, consistent with the biochar nature of the support. The desorption branches do not fully close at very low relative pressures—a phenomenon known as Low-Pressure Hysteresis (LPH)—which is well documented for microporous carbons and arises from kinetic diffusion limitations of N_2_ in narrow micropores at 77 K. Similar behavior has been reported for other biochar materials [60,61]. This behavior is material-intrinsic and does not affect the calculated textural parameters [62].

As listed in Table 2, the spent catalysts have substantially larger specific surface areas and pore volumes than their fresh counterparts. This significant surface area increase likely arises from several concurrent factors: (i) removal of oxygen-containing functional groups during reduction and reaction, as evidenced by the attenuated C-O and O-C=O signals in XPS (Figure 6a) and the diminished -OH/C=O bands in FTIR (Figure 5b); (ii) partial gasification of the carbon support by CO_2_ or H_2_O generated during reaction; (iii) pore opening and structural rearrangement of the biochar matrix under thermal treatment (400 °C reduction +320 °C reaction); (iv) removal of volatile components; and (v) morphological changes induced by the formation and migration of metallic nanoparticles. The partial graphitization observed by HRTEM (0.350 nm graphite fringes) and Raman spectroscopy (decreased I_D_/I_G_ ratio, Supplementary Figure S1) suggests that while some carbon domains become more ordered, the overall pore architecture can still expand—consistent with the measured surface area increases.

The textural properties of our catalysts are comparable to, or better than, those of analogous materials reported in the literature, such as CoFe_2_O_4_, Fe@Co bimetallic composites, and Fe-Co alloy carbides [63,64]. A high specific surface area exposes more active sites, while enlarged pore volume and appropriate pore size facilitate mass transport. The combination of enhanced surface area, hierarchical pore structure (micropores for active site stabilization, mesopores for reactant/product diffusion), and partial graphitization (for improved electron transfer) likely contributes to the favorable catalytic performance we observe [65].

### 3.4. Analysis of Catalytic Process Mechanism

To investigate the atomic-scale mechanism by which material structures influence catalytic performance, we performed density functional theory (DFT) calculations. An Fe_14_Co_4_ alloy cluster derived from bulk Fe_15_Co_5_ was deposited on a graphene (GR) substrate to construct the catalytic model, which consists of 24 carbon atoms, 14 iron atoms, and 4 cobalt atoms. The lattice parameters were set as a = 8.55 Å and b = 7.40 Å, and a 20 Å vacuum slab was introduced to eliminate artificial interactions between periodic images. All calculations were carried out using the Vienna Ab initio Simulation Package (VASP) [66,67]. The Perdew–Burke–Ernzerhof (PBE) generalized gradient approximation was adopted to describe electron exchange and correlation effects [68]. A plane-wave kinetic energy cutoff of 520 eV was utilized, and the Brillouin zone was sampled via a 4 × 4 × 1 K-point mesh. Geometric optimization was terminated when the residual force on each atom fell below 0.02 eV Å^−1^ and the total energy variation converged within 10^−4^ eV. Grimme’s DFT-D3 dispersion correction was incorporated to account for van der Waals interactions [69].

The Gibbs free energy (ΔG) for each elementary step was calculated using the following expression:

(7) $$\Delta G=\Delta E+\Delta E_{\mathrm{ZPE}}-T\Delta S$$

where ΔE is the DFT-calculated electronic energy difference, ΔE_ZPE_ is the zero-point energy correction obtained from vibrational frequency calculations, and ΔS is the entropy change. For the hydrogenation steps, H_2_ dissociation on the metal surface was treated separately, and atomic *H species derived from H_2_ dissociation were used as the hydrogen source. The free energy of the hydrogenation steps was calculated following the thermodynamic framework analogous to the computational hydrogen electrode (CHE) model proposed by Nørskov et al. [70], wherein the chemical potential of a proton–electron pair is referenced to half of a gaseous H_2_ molecule. It should be emphasized that we adopt only the thermodynamic correction framework of the CHE model-the standard approach of correcting DFT electronic energies with zero-point energy and entropic contributions-rather than treating this as an electrochemical system.

The Fe_14_Co_4_/GR model serves as a simplified representation of the complex multiphase compositions (e.g., χ-type carbides, metallic Fe^0^/Co^0^) present in practical catalysts. Its primary purpose is to isolate and elucidate the intrinsic electronic synergy between Fe and Co atoms. The graphene substrate represents the graphitized carbon regions identified in HRTEM, providing an electronically relevant model for the partially graphitized biochar support. The Fe_14_Co_4_ composition corresponds closely to the optimal experimental Fe_15_Co_5_ composition.

The Gibbs free energies were first calculated at 298.15 K and 1 bar. To assess the influence of experimental conditions (593 K, 2 MPa), we performed additional thermodynamic corrections as post-processing steps. Temperature corrections were obtained from vibrational frequency calculations using the formula above. Pressure corrections for gas-phase species were included using $G\left(T,p\right)=G\left(T\right)+RTln\left(p/p_{0}\right)$, where $p_{0}=1$ bar. Detailed energetics at both 298.15 K/1 bar and 593 K/2 MPa are provided in Supplementary Table S3. The qualitative conclusions remain robust at both temperatures and pressures.

Figure 9 illustrates the complete Gibbs free energy pathway for CO_2_ hydrogenation to CH_4_ over the Fe_14_Co_4_/GR model. As shown in the figure, the vertical axis represents the relative Gibbs free energy (G) of each intermediate, with the initial adsorbed state (*+CO_2_) set as the reference point at G = 0.00 eV. The Gibbs free energy change (ΔG) for each elementary step is calculated as the difference between consecutive states (ΔG = G(product)-G(reactant)). CO_2_ adsorbs on Fe-Co bimetallic sites via an η^2^-(C,O) configuration, where the C atom coordinates with Co and one O atom binds to Fe to form the *CO_2_ intermediate (*+CO_2_ at G = 0.00 eV). This adsorption step, followed by subsequent hydrogenation, yields the *COO intermediate (G = −0.825 eV) with a free-energy decrease of −0.825 eV from the gaseous reference, suggesting strong CO_2_ activation by the bimetallic sites. The *COO → *COOH (G = −0.539 eV) step has ΔG = +0.286 eV, and the subsequent *COOH → *CO (G = −0.891 eV) step has ΔG = −0.352 eV. The overall conversion from *COO to *CO is thus thermodynamically favorable when both steps are considered.

Two competing pathways emerge after *CO formation. Direct desorption of CO to gaseous CO (+CO(g), G = +0.728 eV) has ΔG = +1.619 eV (from *CO to CO(g)). Alternatively, *CO hydrogenation produces *COH (G = +0.197 eV) with ΔG = +1.088 eV, followed by conversion to *CHOH (G = +0.632 eV) with ΔG = +0.435 eV. These values represent free-energy differences between stable intermediate states, not kinetic activation barriers. Determining true activation energies would require transition-state calculations. This comparison suggests that Fe-Co bimetallic sites thermodynamically favor the deep hydrogenation pathway over CO desorption at both 298 K and 593 K (Supplementary Table S3), consistent with the experimental observation that the lowest CO selectivity occurs at intermediate Co loading (Fe_15_Co_5_-BC). The transformations *CHOH → *CH_2_OH (ΔG = −0.626 eV) and *CH_2_OH → *CH_2_ (ΔG = −0.337 eV) involve successive O-H bond formation and C-OH bond scission. The hydrogenation steps *CH_2_ → *CH_3_ (ΔG = −0.346 eV) and *CH_3_ → *CH_4_ (ΔG = −0.358 eV) are both exothermic. Methane then desorbs from the catalyst surface, with a pressure-corrected final state of −0.986 eV for *+ CH_4_(g), completing the catalytic cycle.

The geometric configurations of key intermediates along the aforementioned reaction pathway are displayed in Figure 10. The DFT results suggest three aspects of Fe-Co synergy:

Synergistic C-O bond activation. Both initial CO_2_ adsorption and the critical C-O bond cleavage during *COO → *CO rely on concerted electronic modulation by Fe and Co, which thermodynamically facilitates CO_2_ activation—a prerequisite for efficient reaction initiation.

Selective hydrogenation pathway steering. At the *CO branching point, the bimetallic sites stabilize oxygen-containing intermediates, making the deep hydrogenation route (*CO → *COH, ΔG = +1.088 eV) thermodynamically more favorable than CO desorption (*CO → CO(g), ΔG = +1.619 eV). This provides a theoretical rationale for the experimental observation that the lowest CO selectivity occurs at intermediate Fe/Co ratio (Fe_15_Co_5_-BC). The V-shaped CO selectivity trend at higher Co loading may reflect altered surface adsorption properties not captured by the present cluster model.

Global optimization of energy profiles. Apart from the *CO → *COH step, all remaining elementary steps are thermodynamically favorable (exothermic or with small positive ΔG), supporting the overall feasibility of the catalytic cycle from a thermodynamic perspective.

We constructed only the Fe_14_Co_4_ cluster with a fixed atomic ratio in this theoretical study; we did not include models for all experimental Fe/Co ratios. The present model is therefore insufficient to fully explain the composition-dependent selectivity trends across the entire Fe/Co range. The calculations nevertheless suggest that further increasing Co loading could lead to excessive Co occupation of surface active sites, altering the adsorption geometry and stability of reaction intermediates and potentially shifting product selectivity toward C_2+_ hydrocarbons. This inference is consistent with the non-monotonic variation in CH_4_ selectivity observed experimentally. We emphasize, however, that this interpretation remains speculative and would require systematic computational studies with multiple Fe/Co compositions for confirmation. The present theoretical model provides preliminary insights into how Fe/Co atomic ratios may influence product distribution and serves as a foundation for future computational investigations using carbide-phase models with varying Fe/Co ratios.

## 4. Conclusions

In summary, Fe-Co bimetallic catalysts supported on cotton residue-derived biochar were successfully fabricated, and their catalytic performance toward CO_2_ hydrogenation to hydrocarbons was systematically investigated. Characterization indicates that the CoFe_2_O_4_ spinel phase present in the fresh catalyst is essentially transformed into metallic Fe^0^ and Co^0^ after H_2_ reduction. In the subsequent CO_2_ hydrogenation process, some of these metal species were converted into χ-(Fe_1−x_Co_x_) _5_C_2_, which was supported by supplementary XRD, XPS, HRTEM/SAE, and Raman analysis. This conclusion is inferred because the sample was not characterized after H_2_ and before CO_2_ hydrogenation. Fe_15_Co_5_-BC delivered the optimal catalytic performance with a CO_2_ conversion of 49.9% and CH_4_ selectivity of 56.2% within the hydrocarbon fraction, where CH_4_ and C_2_-C_4_ alkanes constituted the dominant hydrocarbon products. This confirms the bifunctional nature of the catalyst system integrating CO_2_ methanation and Fischer–Tropsch hydrogenation activity. The V-shaped CO selectivity trend with Co loading-with the minimum at the intermediate Fe/Co ratio-is primarily attributed to the enhanced overall CO_2_ conversion and efficient conversion of carbon intermediates into hydrocarbon species. Control experiments confirmed that the bimetallic composition is essential for the enhanced performance, with the physical mixture showing only modest improvement over the individual monometallic catalysts. Time-on-stream data further confirmed that steady state was achieved within 120 min, and the catalyst remained stable over the 240 min test period. DFT calculations suggest that the synergistic interaction of Fe-Co bimetallic sites effectively weakens C-O bonds and thermodynamically favors the deep hydrogenation pathway over CO desorption, offering a theoretical rationalization for the tunable product selectivity.

Definitive confirmation of the χ-carbide phase would benefit from additional techniques such as Mössbauer spectroscopy or synchrotron XRD. Long-term stability testing (>500 h) under industrial conditions was not performed in this work. Further in-depth research—including extended time-on-stream testing, post-reaction characterization at multiple time intervals, and quantitative assessment of individual Fe and Co synergistic contributions-will be required to establish the practical viability of these catalysts for industrial applications.

## Figures and Tables

**Figure 1:**
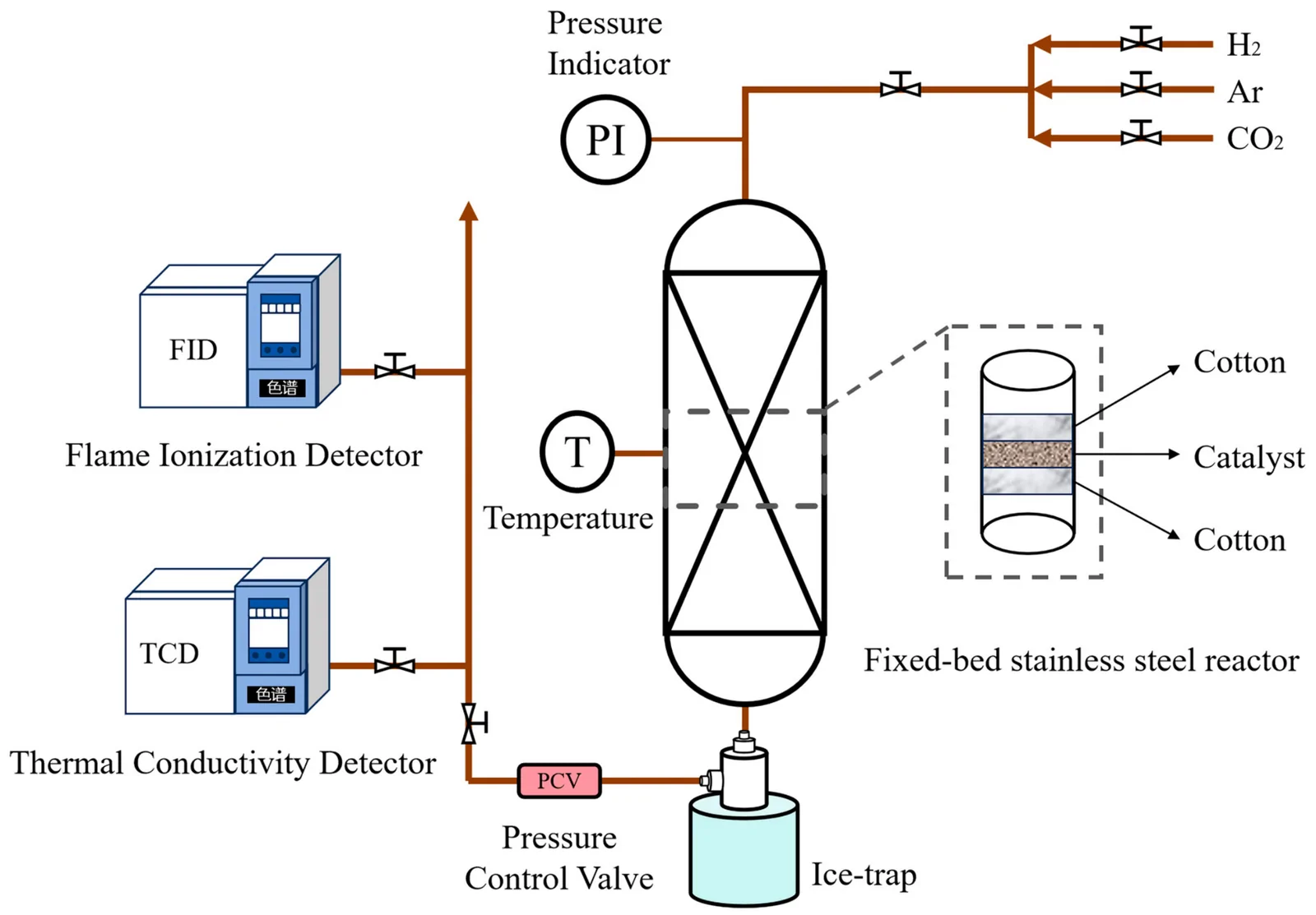
Flow chart of CO_2_ hydrogenation on a fixed bed stainless steel reactor.

**Figure 2:**
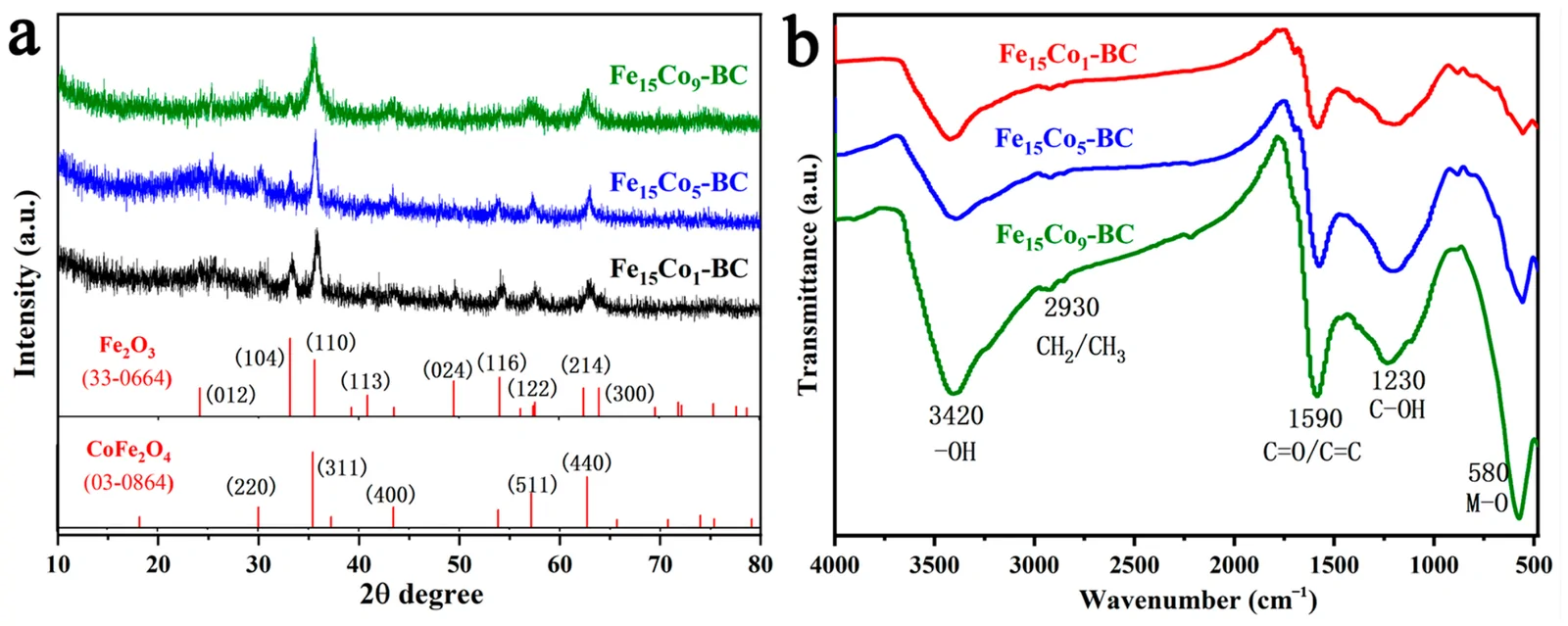
Structure and Composition Characterization. (**a**) XRD Pattern and (**b**) FT-IR of fresh catalysts.

**Figure 3:**
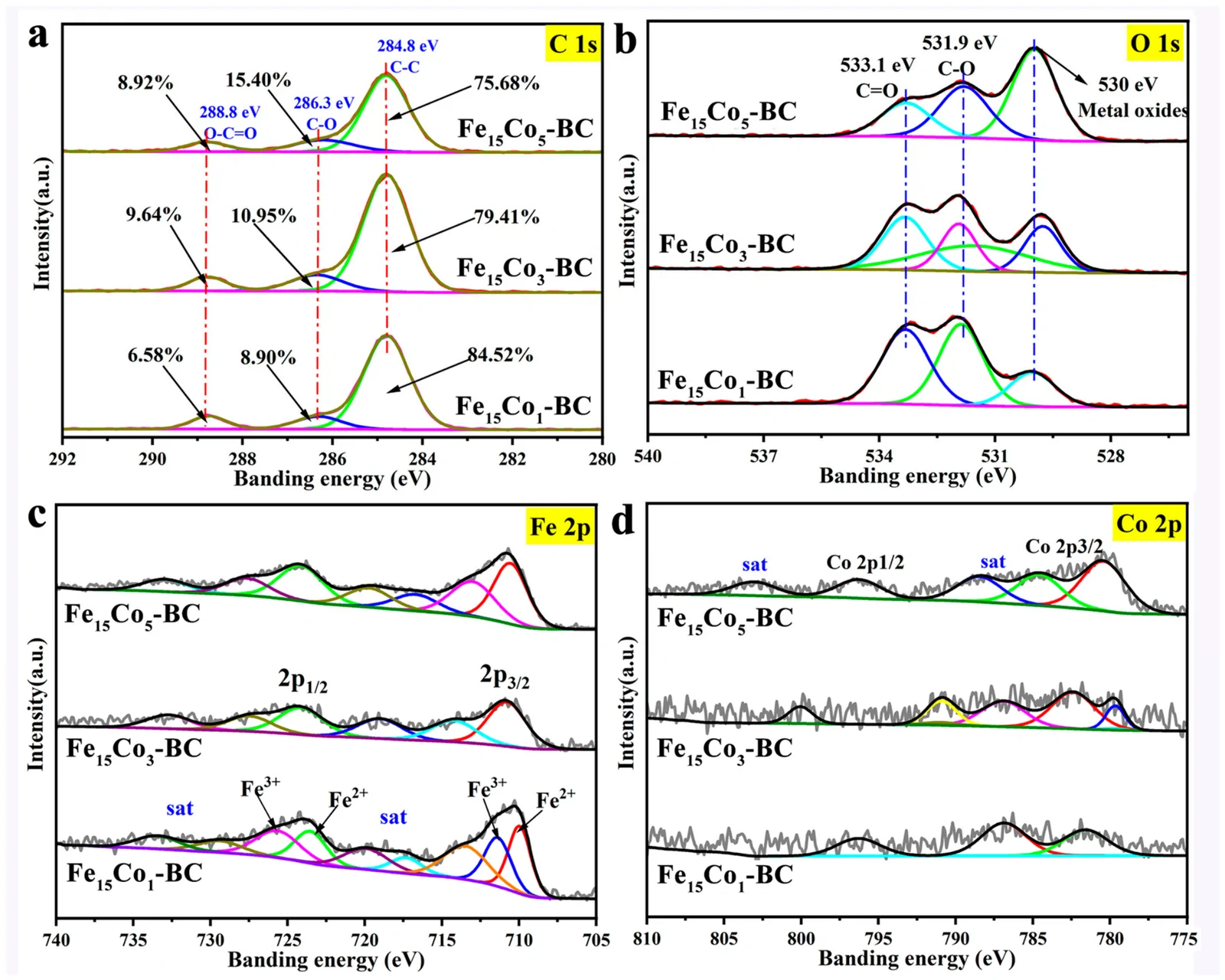
XPS images of three fresh catalysts (**a**) C 1s, (**b**) O 1s, (**c**) Fe 2p, and (**d**) Co 2p.

**Figure 4:**
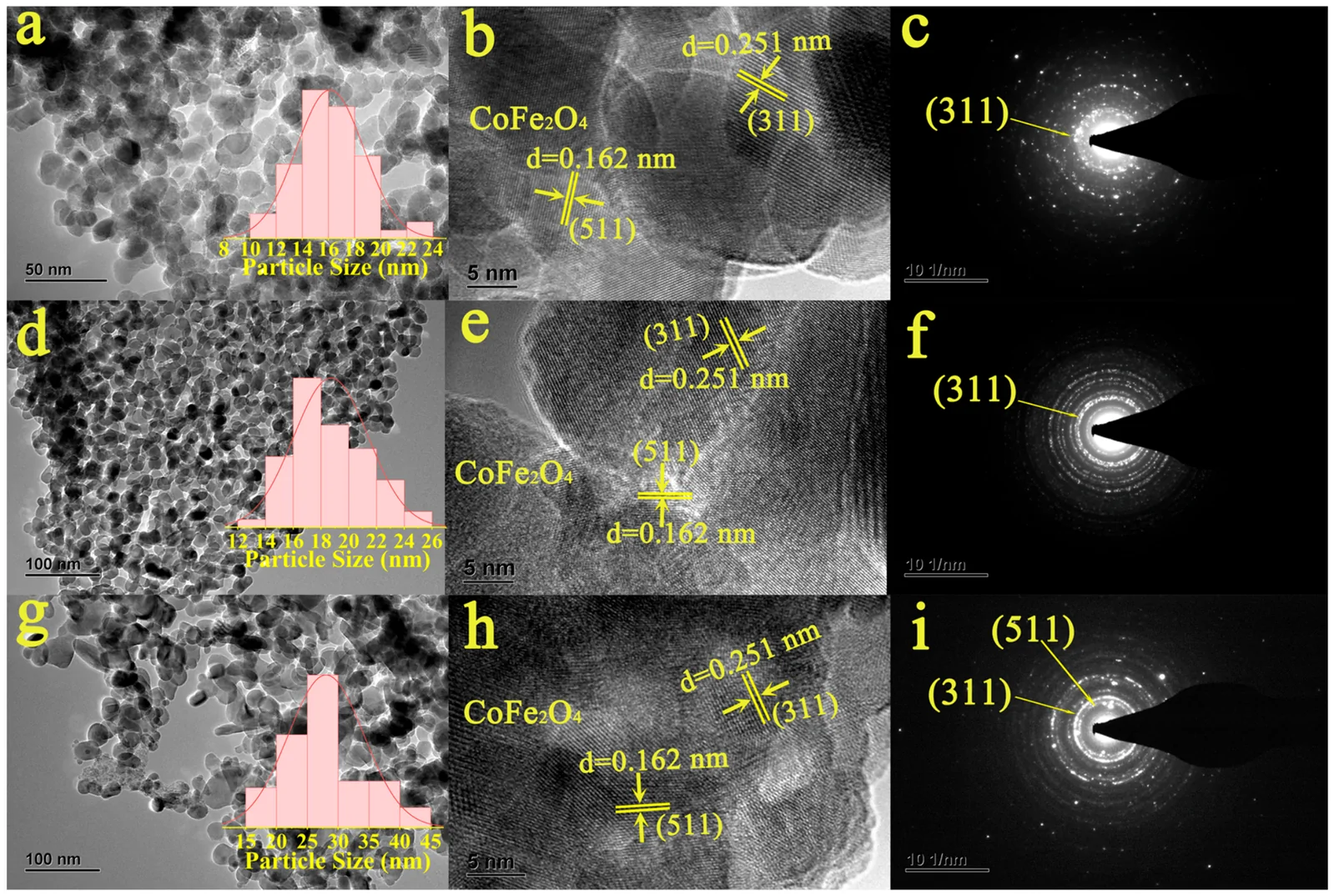
TEM, HRTEM, and SAED images of three fresh catalysts. (**a**–**c**) Fe_15_Co_1_-BC, (**d**–**f**) Fe_15_Co_5_-BC, (**g**–**i**) Fe_15_Co_9_BC.

**Figure 5:**
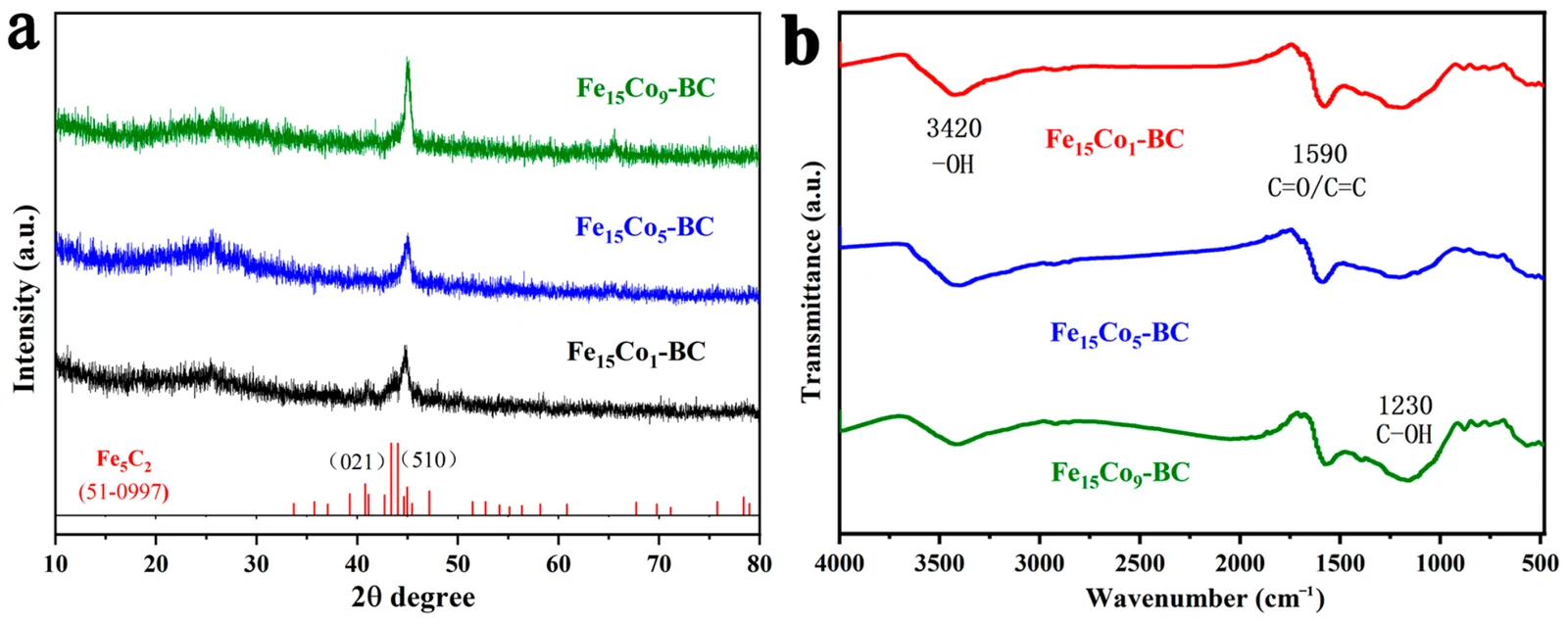
Structural characteristics of spent catalysts. (**a**) XRD patterns of spent catalysts; (**b**) FT-IR spectra of spent catalysts.

**Figure 6:**
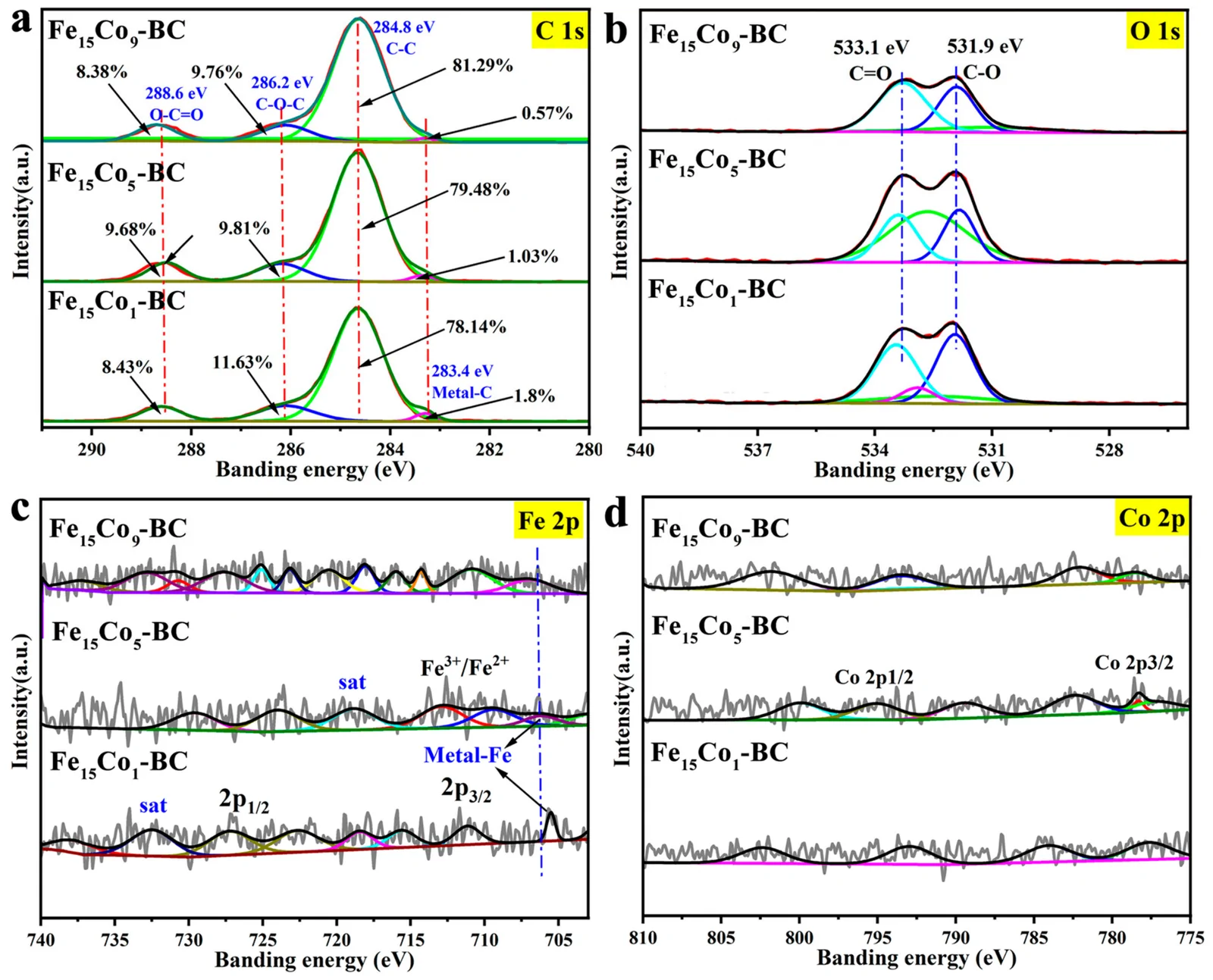
XPS spectra of three spent catalysts. (**a**) C 1s, (**b**) O 1s, (**c**) Fe 2p, and (**d**) Co 2p.

**Figure 7:**
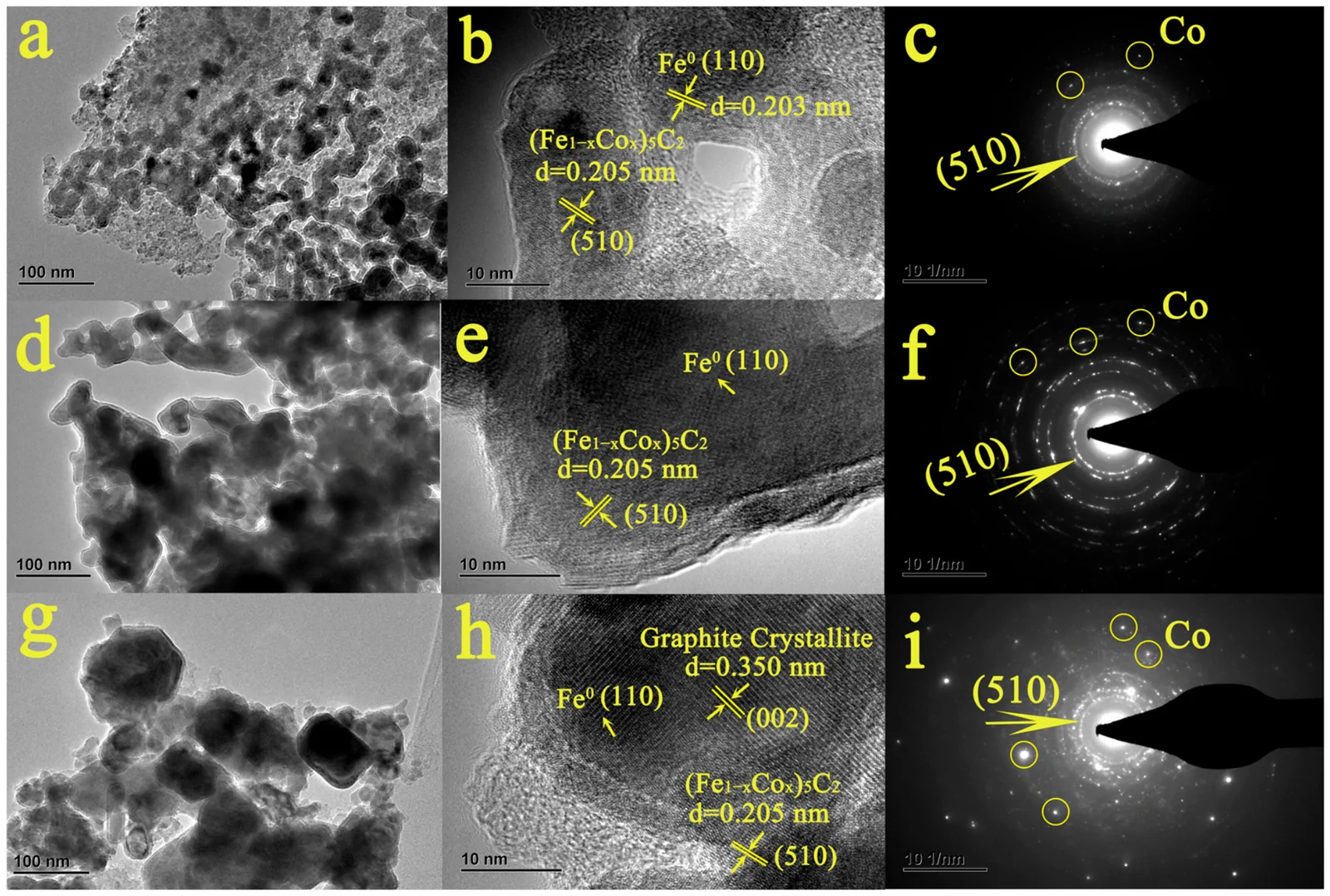
TEM, HRTEM, and SAED images of the three spent catalysts. (**a**–**c**) Fe_15_Co_1_-BC, (**d**–**f**) Fe_15_Co_5_-BC, (**g**–**i**) Fe_15_Co_9_BC.

**Figure 8:**
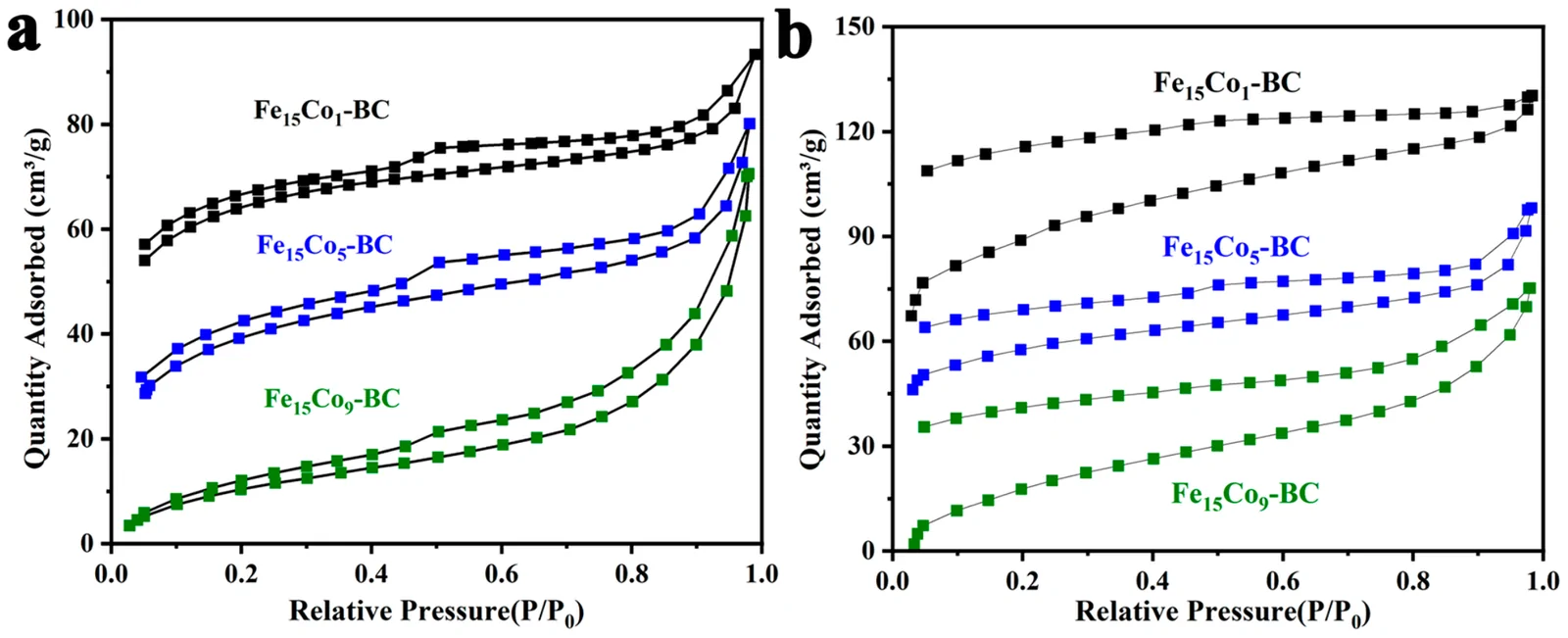
Adsorption–desorption isotherms. (**a**) Three fresh catalysts; (**b**) Three spent catalysts.

**Figure 9:**
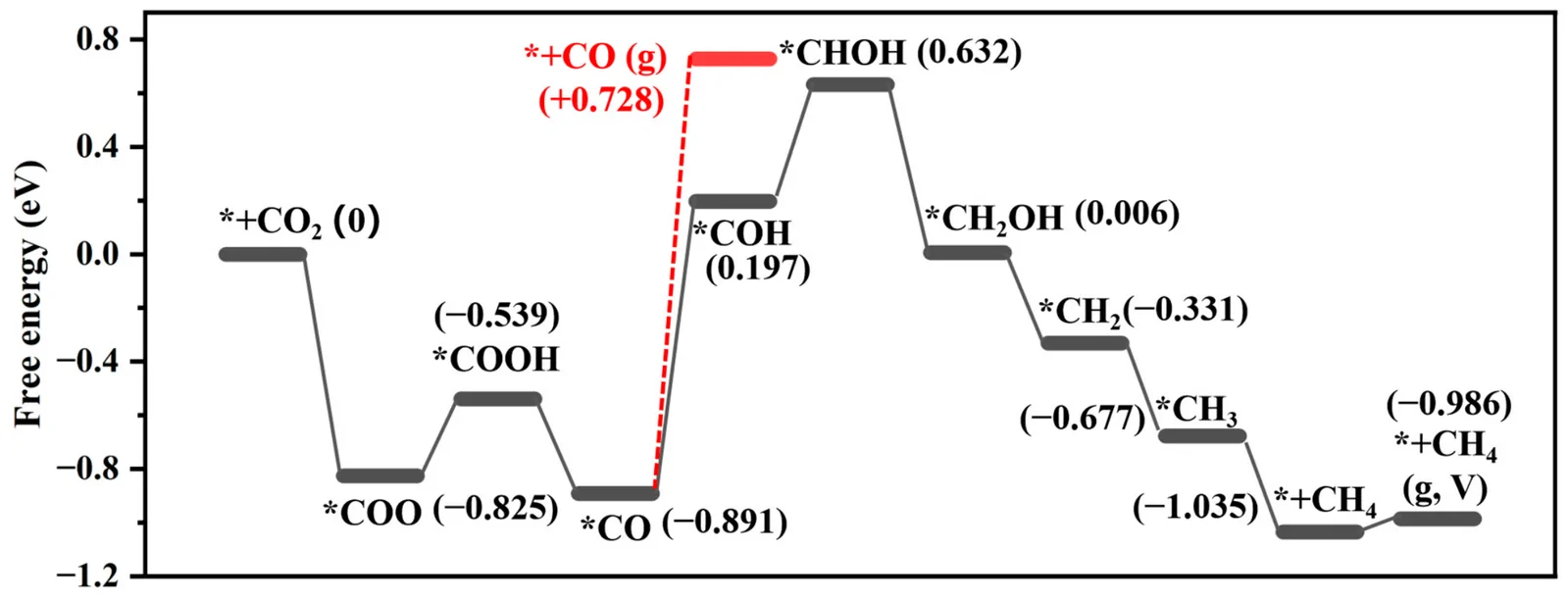
Schematic free energy profile of the complete CO_2_ hydrogenation to CH_4_ over the Fe_14_Co_4_/GR model. The vertical axis denotes the relative Gibbs free energy (G) of each intermediate.

**Figure 10:**
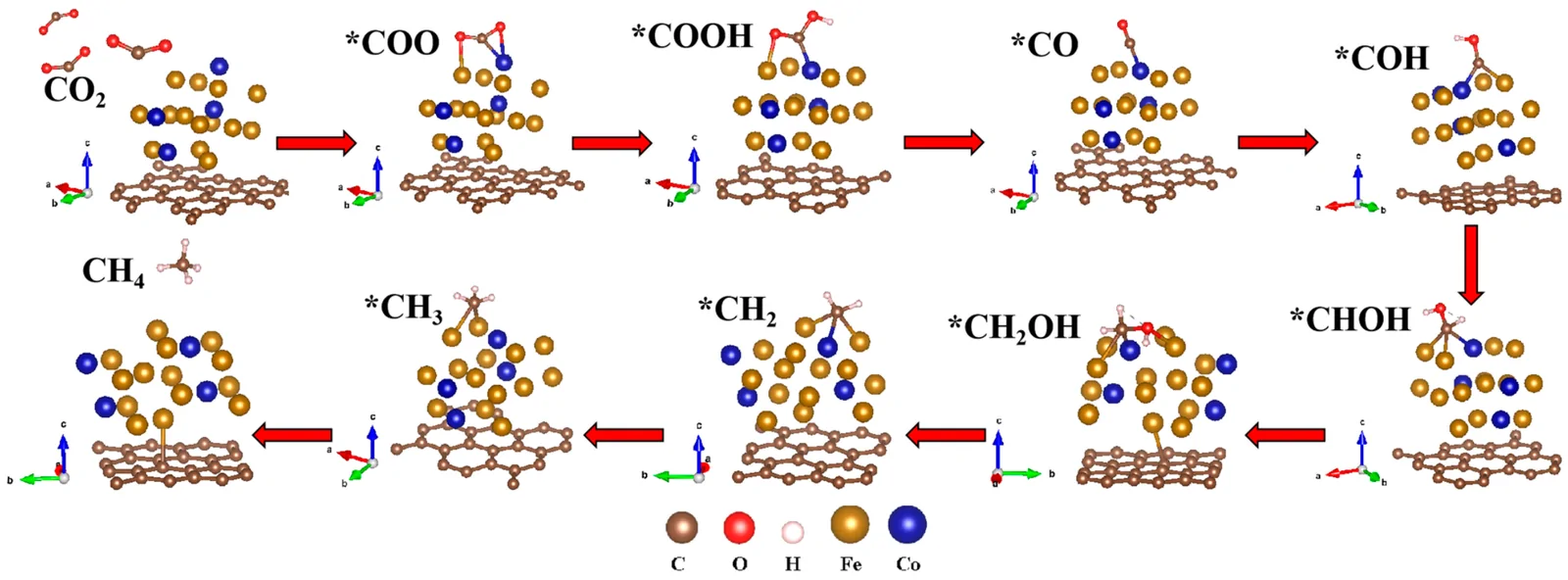
Schematic illustration of the intermediate structures for CO_2_ hydrogenation to CH_4_ on the Fe_14_Co_4_/GR model.

**Table 1:** Catalytic performance of all catalysts for CO_2_ hydrogenation.

Catalyst	CO_2_ Conv. (%)	CO Sel. (%)	Total HC Sel. (%)	HC Yield (%)	Carbon Balance (%)	CH_4_ (%)	C_2_-C_4_ = (%)	C_2_-C_4_ (%)	C_5+_ = (%)	C_5+_ (%)
Biochar	<1	-	-	-	-	-	-	-	-	-
Fe_15_-BC	28.3	44.5	55.5	15.7	97.9	18.7	1.5	42.3	6.8	30.7
Co_15_-BC	32.1	41.2	58.8	18.9	98.0	22.3	0.8	45.1	5.2	26.6
Fe_15_-BC + Co_15_-BC	35.6	38.7	61.3	21.8	97.8	25.1	1.2	43.8	5.8	24.1
Fe_15_Co_1_-BC	37.8	27.2	72.8	27.5	98.2	38.5	0.9	39.6	4.1	16.9
Fe_15_Co_5_-BC	49.9	19.6	80.4	40.1	98.6	56.2	0.1	41.0	0.3	2.4
Fe_15_Co_9_-BC	44.7	28.8	71.2	31.8	97.6	46.7	0.4	39.6	2.2	11.1

Notes: (1) Reaction conditions: 320 °C, 2.0 MPa, H_2_/CO_2_ = 4:1, GHSV = 6000 mL·g^−1^·h^−1^, time-on-stream = 4 h. (2) Calculate the relevant results using Formulas (1)–(6). (3) Hydrocarbon distribution columns (CH_4_ through C_5+_) are normalized within total hydrocarbons; each row sums to 100%. (4) C_2_-C_4_=: C_2_-C_4_ olefins; C_2_-C_4_: C_2_-C_4_ alkanes; C_5+_=: C_5+_ olefins; C_5+_: C_5+_ alkanes.

**Table 2:** The BET results for the different catalysts.

Catalysts	Specific Surface Area (m^2^/g)	Pore Volume (cm^3^/g)	Average Pore Size (nm)
f-Fe_15_Co_1_-BC	64.994	0.072	2.211
f-Fe_15_Co_5_-BC	141.677	0.118	1.304
f-Fe_15_Co_9_-BC	86.374	0.126	1.173
s-Fe_15_Co_1_-BC	326.548	0.176	1.158
s-Fe_15_Co_5_-BC	163.798	0.120	1.178
s-Fe_15_Co_9_-BC	224.032	0.169	1.187

Note: f represents fresh, s represents spent. Average pore size was calculated by the BJH method from the desorption branch.

## Data Availability

The original contributions presented in this study are included in the article/Supplementary Materials. Further inquiries can be directed to the corresponding author.

## Supplementary Materials

The following supporting information can be downloaded at: https://www.mdpi.com/article/10.3390/ma19194034/s1, Table S1. ICP-OES Results of Prepared Catalysts. Table S2. Time-on-stream data for Fe_15_Co_5_-BC catalyst at 320 °C. Table S3. Comparison of relative Gibbs free energies (G, eV) of key intermediates and corresponding elementary step free energy changes (ΔG, eV) for CO_2_ hydrogenation to CH_4_ over Fe_14_Co_4_/GR model at different temperatures and pressures. Figure S1. Raman spectra of fresh and spent Fe_15_Co_5_-BC catalysts.
