# Supplementary material for: Synthesis of Fe-Co Bimetallic Supported on Cotton Residue Biochar: Catalytic Performance and Mechanistic Investigation for CO2 Hydrogenation
Source: Materials (Basel). 2026 Sep 22;19(19):4034. doi: 10.3390/ma19194034 (PMC13666206; doi:10.3390/ma19194034)
Supplement: Supplementary file 1 [file materials-19-04034-s001.zip › materials-4555970-supplementary.pdf]

Supplementary information

Synthesis of Fe-Co bimetallic supported on cotton residue biochar: catalytic performance and mechanistic investigation for CO_2 hydrogenation

Youzhi Yao[1], Tiancheng Yao[2], Qinghua Deng[1], Jiaxin Jiang[1], Tianyu Wang[3]，Wenjie Wu[1*]

([1]College of Materials Engineering, Wuhu Vocational Technical University, Wuhu 241003, China

[2]College of Materials Science and Engineering, Hohai University, Changzhou 213000, China

[3]Key Laboratory of Environmental Remediation and Ecological Health, Ministry of Industry and Information Technology, School of Environmental and Biological Engineering, Nanjing University of Science and Technology, Nanjing, 210094, China

Corresponding author: E-mail addresses: wuwenjie21@mails.ucas.ac.cn

Table S1. ICP-OES Results of Prepared Catalysts

Catalyst	Fe Loading (wt%)	Co Loading (wt%)	Total Metal Loading (wt%)	Nominal Fe/Co (molar)	Measured Fe/Co (molar)
Fe_{15}-BC	**14.8**	—	**14.8**	15:0	—
Co_{15}-BC	—	**15.1**	**15.1**	0:15	—
$Fe_{15}Co_1$-BC	**14.2**	**1.0**	**15.2**	15:1	**14.7:1.1**
$Fe_{15}Co_5$-BC	**12.8**	**4.3**	**17.1**	15:5 (3:1)	**14.9:4.8**
$Fe_{15}Co_9$-BC	**11.5**	**7.6**	**19.1**	15:9 (5:3)	**14.6:9.2**

Table S2. Time-on-stream data for $Fe_{15}Co_5$-BC catalyst at 320 °C

Time-on-Stream (min)	CO_2 Conversion (%)	CH_4 Selectivity (%)	CO Selectivity (%)	C_2-C_4 Alkanes Selectivity (%)
0	—	—	—	—
30	45.2	50.8	23.5	39.2
60	48.5	54.2	20.8	40.5
90	49.3	55.6	20.1	40.8
**120**	**49.8**	**56.0**	**19.8**	**41.0**
**150**	**50.0**	**56.3**	**19.6**	**41.1**
**180**	**49.7**	**56.0**	**19.7**	**41.0**
**210**	**49.9**	**56.4**	**19.5**	**41.2**
**240**	**50.1**	**56.2**	**19.6**	**41.0**
**Average (120–240 min)**	**49.9 ± 0.5**	**56.2 ± 0.6**	**19.6 ± 0.4**	**41.0 ± 0.5**

Note: Selectivities for C_2-C_4 olefins and C_{5+} olefins are omitted for clarity; these remained stable at 0.1–0.3% throughout the test. The C_{5+} alkane selectivity remained stable at approximately 2.4%.

Table S3. Comparison of relative Gibbs free energies (G, eV) of key intermediates and corresponding elementary step free energy changes (ΔG, eV) for CO_2 hydrogenation to CH_4 over $Fe_{14}Co_4$/GR model at different temperatures and pressures.

Intermediate / Elementary Step	G (298.15 K, 1 bar) / eV	G (593.15 K, 1 bar) / eV	G (593.15 K, 2 MPa) / eV	ΔG (298.15 K, 1 bar) / eV	ΔG (593.15 K, 1 bar) / eV	ΔG (593.15 K, 2 MPa) / eV
* + CO_2 (reference)	0.000	0.000	0.000	—	—	—
*COO	-0.825	-0.701	-0.701	—	—	—
*COOH	-0.539	-0.429	-0.429	—	—	—
*CO	−0.891	−0.718	−0.718	—	—	—
*COH	+0.197	+0.435	+0.435	—	—	—
*CHOH	+0.632	+0.879	+0.879	—	—	—
* + CO(g)	+0.728	+0.552	+0.705	—	—	—
*CH_2OH	+0.006	+0.171	+0.171	—	—	—
*CH_2	-0.331	-0.177	-0.177	—	—	—
*CH_3	-0.677	-0.527	-0.527	—	—	—
* + CH_4	-1.035	-0.892	-0.892	—	—	—
* + CH_4 (g, V)	-0.986	-0.843	−0.690	—	—	—
**Elementary Steps (ΔG = G(product) − G(reactant))**						
* + CO_2 → *COO	—	—	—	-0.825	-0.701	-0.701
*COO → *COOH	—	—	—	+0.286	+0.272	+0.272
*COOH→ *CO	—	—	—	-0.352	-0.289	-0.289
*CO → * + CO(g) (desorption)	—	—	—	+1.619	+1.270	+1.423
*CO → *COH	—	—	—	+1.088	+1.153	+1.153
*COH → *CHOH	—	—	—	+0.435	+0.444	+0.444
*CHOH → *CH_2OH	—	—	—	-0.626	-0.708	-0.708
*CH_2OH → *CH_2 + *OH	—	—	—	-0.337	-0.348	-0.348
*CH_2 + *H → *CH_3	—	—	—	-0.346	-0.350	-0.350

Intermediate / Elementary Step	G (298.15 K, 1 bar) / eV	G (593.15 K, 1 bar) / eV	G (593.15 K, 2 MPa) / eV	ΔG (298.15 K, 1 bar) / eV	ΔG (593.15 K, 1 bar) / eV	ΔG (593.15 K, 2 MPa) / eV
$*CH_3 + *H \rightarrow * + CH_4$	—	—	—	-0.358	-0.365	-0.365
$*+CH_4 \rightarrow *+CH_4$ (g,V)	—	—	—	+0.049	+0.049	+0.202

Table Notes:

Temperature correction: Values at 593.15 K are calculated using ΔG(593 K) = ΔH − 593·ΔS, with $\Delta H \approx \Delta E + \Delta E_{ZPE}$ obtained from vibrational frequency calculations.

Pressure correction: For gas-phase species (CO_2, H_2, CO, CH_4), pressure correction is applied using $G(T,p) = G(T) + RT\ln(p/p_0)$ with p_0 = 1 bar and p = 20 bar (2 MPa), giving $RT\ln(20) \approx 0.078$ eV per gas-phase molecule at 298.15 K and ≈0.153 eV at 593.15 K. This correction is included in columns 3, 4, 6, and 7 as appropriate.

Figure S1. Raman spectra of fresh and spent $Fe_{15}Co_5$-BC catalysts

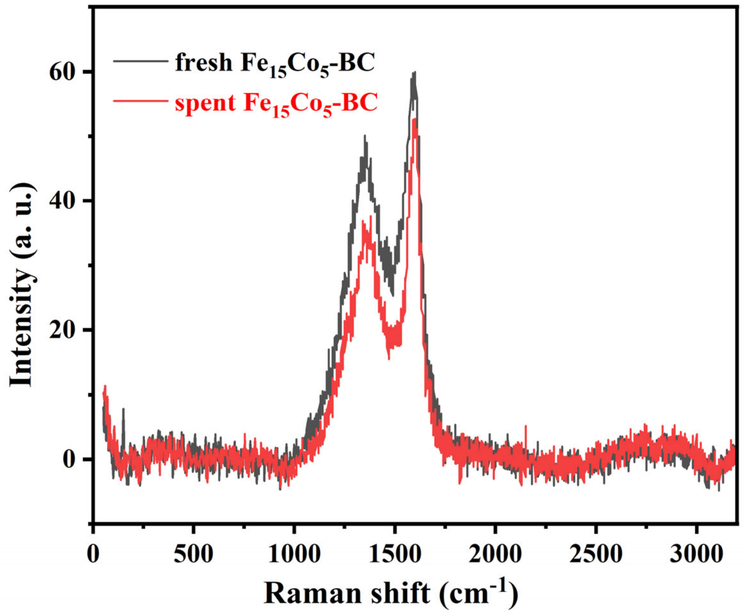

Raman spectra of fresh and spent $Fe_{15}Co_5$-BC catalysts. The D band (defect-induced, ~1350 cm-1) and G band (graphitic, ~1580 cm-1) are labeled. After peak fitting with Lorentzian functions, the I_D/I_G area ratio decreases from 2.14 (fresh) to 1.98 (spent), and the I_D/I_G height ratio decreases from 0.88 to 0.70, suggesting partial graphitization of the biochar support during the CO_2 hydrogenation reaction. The decrease in I_D/I_G ratio indicates an increase in the degree of graphitization of the carbon support.
